# Extracellular Virions: The Advance Guard of Poxvirus Infections

**DOI:** 10.1371/journal.ppat.1004904

**Published:** 2015-07-02

**Authors:** David J. Pickup

**Affiliations:** Department of Molecular Genetics and Microbiology, Duke University, Durham, North Carolina, United States of America; University of Florida, UNITED STATES

## Extracellular Virions (EVs) Are the Most Highly Processed Poxvirus Particles

Most poxviruses produce multiple types of virions: intracellular mature virions (MVs), wrapped virions (WVs), and extracellular virions (EVs). The EVs represent the culmination of a morphogenetic pathway including the development of MVs, which are then transformed into WVs, and finally transformed into EVs. Each EV consists of one form of MV wrapped within a second lipid membrane containing at least four, and usually six, EV-specific viral proteins [reviewed in 1,2]. Although EVs are the end product of the viral morphogenetic pathway they often comprise <1% of the total progeny virions [3]. The conservation of the ability to produce EVs amongst most poxviruses indicates that EVs are generally advantageous for poxvirus replication in vivo. While EVs are not essential for virus infection either in vitro or in vivo, viruses lacking the capacity to efficiently produce EVs are usually highly attenuated in vivo [reviewed in 4]. These attributes suggest that EVs have specialized roles distinct from those of infectious MVs in viral replication.

## EVs Promote Their Own Release and Extracellular Transport

The WVs are produced from intracellular MVs by the addition of two lipid membranes. The WVs are actively transported via microtubules to the plasma membrane, where fusion of the outermost WV membrane with the plasma membrane allows the WV to be released onto the surface of the cell as an EV [reviewed in 2,5]. This mechanism allows the EVs to leave the infected cell with minimal corelease of materials that might activate host defenses. Once on the surface of the cell, the EVs may be rapidly propelled on the tips of actin-rich projections across the surfaces of infected cells [reviewed in 2]. This extraordinary form of extracellular transport is controlled by EV proteins that induce infected cells to form the actin-filled projections that are capable of repeatedly pushing the EVs away from infected cells [reviewed in 6–8]. This process can accelerate the spread of the virus from an infected cell to surrounding uninfected cells as evidenced by the presence of EVs far ahead of the expanding perimeter of productively infected cells in a viral plaque [7].

## EVs Are Important for the Dissemination of Virus in the Host

Early studies suggested that EVs help the spread of the virus within the host [9,10]. This spread is thought to be largely cell-associated, either through the trafficking of motile cells infected by EVs or through the trafficking of motile cells carrying infectious EVs on their surfaces. In the latter case, the active release of EVs from the cell, together with mechanisms to suppress viral reinfection or superinfection of cells, can also result in the accumulation of infectious cell-associated EVs (CEVs) on the surface of infected cells [11]. In addition, vaccinia virus infection itself can induce the migration of the infected cells [12].

The EVs possess a number of properties that help them to disseminate within the host. In particular, the EVs are relatively resistant to immune attack. Their outer membrane masks the surface membrane of the internal MV, rendering the EV resistant to neutralizing antibodies targeting up to five MV surface proteins. Although the EV has up to six viral proteins in its outer membrane, only one of these, the B5 protein, is the target of EV-neutralizing antibodies in vaccinated humans [13]. Further, the EV itself is resistant to antibody-mediated neutralization except in the presence of complement, which may be impaired in its ability to neutralize this form of the virus because of the inclusion of host complement control proteins in the EV membrane [reviewed in 2].

## EVs Suppress Immune Responses to Infection

Viral dissemination within the host influences viral pathogenicity. It also influences host immune responses through the distribution of infectious virus to cells that normally provide functions critical to immune defenses. For example, orthopoxviruses such as vaccinia and cowpox viruses each encode multiple proteins capable of suppressing or affecting of innate and adaptive immune responses [reviewed in 14,15]. And among primary human hematolymphoid cells, vaccinia virus preferentially infects dendritic cells, as well as monocytes, macrophages, B cells, and activated T cells [16], where the expression of viral proteins may be sufficient to affect cellular functions even if the infection is abortive [17].

New evidence that EVs can play a direct role in affecting immune responses has come from one of the oldest available systems for the study of virus–host interactions, the culture of viruses in the chorioallantoic membranes (CAMs) of developing chick embryos. The formation of intense red pocks by cowpox virus in CAMs provides one of the most vivid examples of viral suppression of host innate immune responses. Spontaneous deletion mutants of cowpox virus were found to produce raised opaque white pocks generated primarily by a massive influx of heterophils and macrophages [18], which play critical roles in the immune defense of the host [19]. The white pocks contained decreased levels of virus antigen and lower viral loads in comparison to red pocks produced by wild-type cowpox virus [20]. In sharp contrast to the white pocks, the red pocks are characterized by the minimal presence of heterophils and macrophages, as well as vasodilation (providing the red color) beginning within 48 hours of infection and hemorrhage becoming evident later in the infection [18,20]. Thus, the wild-type cowpox virus encodes proteins capable of suppressing innate immune responses that generate white pocks and concomitantly restrict viral replication within the CAM. At least some of the factors contributing to this suppression appear to be unique to cowpox virus because its pocks are distinct from those of other viruses, including closely-related poxviruses, such as specific rabbitpox and vaccinia viruses, which produce pocks having characteristics intermediate between those of the wild-type and deletion mutants of cowpox virus [21].

The first viral gene to be identified as necessary for red pock formation was the *crmA* gene, which encodes a potent inhibitor of caspase-1, enabling the virus to inhibit the activation and secretion of the proinflammatory cytokines interleukin-1 and interleukin-18 [22,23]. While this finding explained the powerful viral suppression of inflammation in red pocks to some extent, it was clear that additional viral genes are required to effect red pock formation [22,23].

Surprisingly, the second viral gene identified as necessary for red pock formation, the *B5R* gene, did not encode a protein known to affect components of immune responses, but rather the B5 protein present on the surface of EVs, as well as a shorter secreted version of this protein, both sharing some similarity with complement control proteins. Rabbitpox virus mutants lacking this gene produced white pocks containing the massive influx of heterophils typical of pocks produced by *crmA* deletion mutants of cowpox virus [24]. The apparent similarity of the white pocks produced by these two mutations suggested that the B5 protein might also affect inflammatory processes. However, qualitative differences between white pocks of the two types have been identified: dexamethasone treatment fails to suppress delta *B5R* white pock formation, and heterophils in these pocks do not appear to be activated [25]. These findings, together with known host range restrictions and the impaired ability of *B5R* deletion mutants to produce EVs [26], suggested that some form of viral replication deficiency might contribute to the production of white pocks by the *B5R* deletion mutants [25]. However, the precise reason why deletion of the *B5R* gene contributed to the influx of heterophils to the site of viral infection remained unclear.

Recently, Xu and colleagues [27] have brought new information to bear on this issue. Using a library of 109 single-gene knockout variants of cowpox virus, in addition to the *crmA* and *B5R* genes, they identified 10 nonessential genes that are required for the production of red pocks by cowpox virus. These genes are the homologs of the vaccinia virus genes encoding the F12, F13, F15, E2, E3, E8, A4, A33, A34, and A36 proteins. Deletion of the homolog of the vaccinia virus gene encoding the F13 protein failed to generate any pocks (red or white) on the CAM. Significantly, six of these proteins (Table 1), together with the B5 protein, correspond to the viral proteins necessary for the efficient production and transport of the EVs [reviewed in 2,4,5,8]. The one unexplained absence from this list is the *A27L* gene, whose deletion diminishes EV production by vaccinia virus [reviewed in 2,4]. Nonetheless, the requirement for each of the other proteins indicates that for cowpox virus to suppress innate immune responses leading to the formation of white pocks, the virus requires not only viral proteins that target immune responses and control host range, but also the EVs themselves, as well as the actin-mediated transport of the EVs from infected cells.

**Table 1:** Vaccinia virus proteins involved in the formation and transport of EVs [reviewed in 2,4,5,8].

**Protein**	**Roles**
****A27****	Contributes to the formation and microtubule-mediated transport of the WVs.
****A33**** *	Affects the incorporation of the B5 and A34 proteins into the outer membrane of EVs.
	Required for the formation of actin-filled projections; required (with A36 proteins) on the surface of infected cells to interact with B5 proteins on EVs to promote actin-mediated transport of EVs.
****A34**** *	Loss of A34 protein results in a severe reduction in the number of WVs formed.
	Affects EV release from cells and EV infectivity.
	Loss of the A34 protein increases the resistance of the outer membrane of EV to spontaneous rupture.
	Promotes the incorporation of the B5 protein into the WV/EV.
****A36**** *	Loss of A36 protein reduces the efficiency of WV transport to the plasma membrane.
	Required for the formation of actin-filled projections; required (with A33 proteins) on the surface of infected cells to interact with B5 proteins on EVs to promote actin-mediated transport of EVs.
****B5**** *	Loss of B5 protein reduces the numbers of WVs formed.
	Required for the formation of actin-filled projections; required to activate Src and phosphorylate the A36 protein to enable actin polymerization.
****E2**** *	Forms a complex with the F12 protein.
	Involved in WV morphogenesis.
	Involved in microtubule-mediated transport of WVs to the plasma membrane.
	Loss of the E2 protein results in a significant reduction of EV formation.
****F11****	Through inhibition of RhoA signaling, F11 protein promotes cell motility and viral morphogenesis, as well as remodeling of cortical actin to facilitate virus transport to the plasma membrane. F11 protein promotes virus release from the cell, but this protein is not required for the production of EVs.
****F12**** *	Forms a complex with the E2 protein.
	Forms a complex with the A36 protein.
	Involved in WV morphogenesis.
	Involved in microtubule-mediated transport of WVs to the plasma membrane.
	Loss of the F12 protein results in a significant reduction of EV formation.
****F13**** **	Required for WV formation.
	(The F13 protein is the target of the antipoxviral drug ST-246).

* Cowpox virus mutants lacking the ability to encode these proteins produced white pocks on the CAM [27].

**A cowpox virus mutant lacking the ability to encode the F13 protein failed to produce any pocks on the CAM [27].

## EVs in a New Light

**Fig 1:**
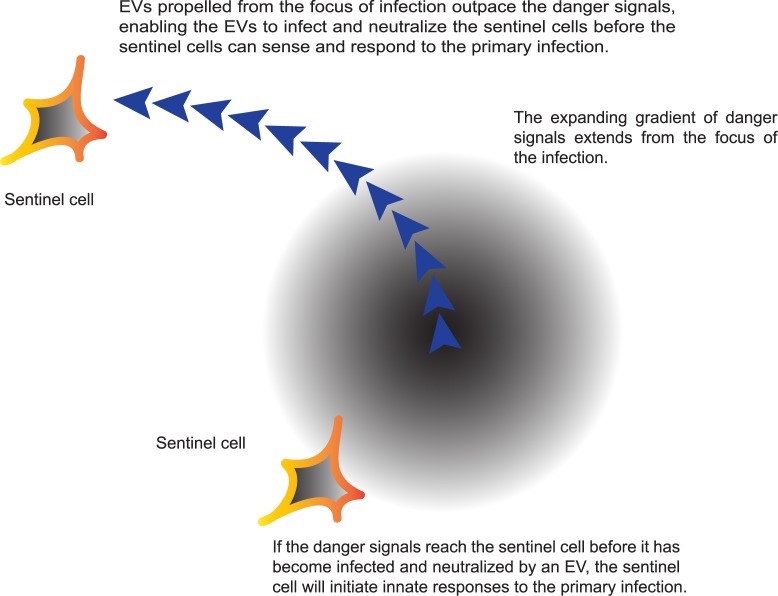
The advance guard model of poxvirus infection. EVs are actively released from intact infected cells and propelled from those cells to outpace the spread of antiviral cytokines, interferons, proinflammatory cytokines, PAMPs, and DAMPs, which are subsequently released from infected cells, and which may initiate innate immune responses from resident or infiltrating sentinel cells. This mechanism may also enable productive infections of neighboring cells that might otherwise be rendered refractive to viral replication by interferons and danger signals released from cells at the site of the primary infection. Cell-associated EVs carried long distances from the primary site of infection by motile cells may similarly be able to initiate infections in the absence of accompanying danger signals.

Collectively, these findings suggest that EVs may contribute to the suppression of immune responses by acting as an advance guard, moving ahead of the main body of infectious progeny viruses to infect and neutralize immune cells (resident cells or cells infiltrating the infected tissue) before these cells can detect and respond to danger signals originating from the site of the primary infection. The danger signals might include interferons, proinflammatory cytokines, or pathogen-associated and damage-associated molecular patterns (PAMPs and DAMPs). Thus, as appears to be the case in the red pocks produced in the CAMs [18,20], virus replication in the tissue can proceed with little to no immediate recognition or response by the host. In this model (Fig 1), the effectiveness of the EV-mediated immune suppression depends on: (i) the ability of the EVs to neutralize a targeted cell by expressing appropriate viral factors to control immune responses, and (ii) the capacity of the EVs to reach and infect the targeted cell. If the EVs can suppress immune responses before that cell can sense and respond to the initial infection, this would provide one explanation for the advantage the virus may derive from the formation of EVs and their actin-mediated transport from the infected cell.

This advance guard model of EV-mediated immunosuppression has several practical implications. It suggests that drugs such as ST-246 [28], which act by inhibiting EV formation, should contribute to the control of poxvirus infections not only by restricting the dissemination of the virus but also by reducing the capacity of the virus to suppress immune defenses to infection. Similarly, the protective effects of antiorthopoxvirus vaccines targeting components of the EVs, including the B5 protein, may be partly the result of interference with EV-mediated suppression of immune defenses. Lastly, poxvirus vaccine vectors that cannot produce EVs, either because of viral replication deficiency or because of a defect in a gene needed for EV formation, may benefit from a lack of EV-mediated suppression of immune responses towards these vaccines.
